# Diagnostic Dilemmas: A Review of Reported Cases of Human Herpesvirus 6 Encephalitis in Immunocompetent Adults

**DOI:** 10.1093/ofid/ofae501

**Published:** 2024-09-04

**Authors:** Gemma Webb, Mei Yen Michelle Leong, Emma Bishop, Marjoree Sehu

**Affiliations:** Monash University, Victoria, Australia; Department of Infectious Diseases, Frankston Hospital, Peninsula Health, Victoria, Australia; Monash University, Victoria, Australia; Department of Infectious Diseases, Frankston Hospital, Peninsula Health, Victoria, Australia; Department of Infectious Diseases, Frankston Hospital, Peninsula Health, Victoria, Australia; University of Queensland, Queensland, Australia

**Keywords:** human herpesvirus 6, encephalitis, immunocompetent, polymerase chain reaction

## Abstract

Human herpesvirus 6 (HHV-6) is associated with its presentation in the pediatric population as roseola infantum. Rarely, it is the causative agent of encephalitis, with most cases reported among the immunocompromised population due to reactivation. This review article analyzes the published records of cases labeled HHV-6 encephalitis in immunocompetent adults, aiming to understand the diagnostic methods behind each case and explore the complexities of such a diagnosis. We note significant variability in the methods used to come to a diagnosis of HHV-6 encephalitis, as well as inconsistent approaches to treatment of this condition. Given the rarity of HHV-6 encephalitis in immunocompetent adults, there are no clearly structured diagnostic guidelines for this condition in this patient population. We highlight several diagnostic methods that provide more convincing evidence of true HHV-6 encephalitis and may provide a basis for further development of guidelines for the diagnosis and treatment of this condition.

## Teaser

Human Herpesvirus-6 Encephalitis is exceedingly rare amongst the immunocompetent population. This review article identifies the available records of HHV-6 encephalitis in this demographic to better understand and evaluate the variable diagnostic methods and management of this condition.

Human herpesvirus 6 (HHV-6) is a member of the roseolovirus genus and a part of the herpesvirus family. Its clinical presentation has been noted predominantly in its action as the causative agent of *Roseola infantum*, a relatively innocuous childhood infection. It has increasingly been identified as a causative agent of encephalitis in immunocompromised patients, largely due to viral reactivation [1, 2]. As multiplex polymerase chain reaction (PCR), quantitative PCR, and other diagnostic tools become more available to clinicians, HHV-6 DNA has been identified in the cerebrospinal fluid (CSF) of immunocompetent patients presenting with otherwise unexplained clinical findings consistent with an encephalitis presentation.

An example of such a case was a 67-year-old man who presented to an Australian metropolitan emergency department (ED) after experiencing a generalized tonic-clonic seizure at home on a background of 1 week of vomiting and general malaise for 1 month. Notably, the patient had no headache, neck stiffness, photophobia, paresthesia, or loss of limb power. Lumbar puncture (LP) on D3 revealed mild hyperglycorrhachia, elevated protein (0.76 g/L), a leukocyte and erythrocyte count of 0 cells/L, and negative cytology. Infective tests performed on the patient’s CSF such as gram stain and culture, Herpes Simplex Virus 1, Herpes Simplex Virus 2, Varicella Zoster Virus, and Cytomegalovirus PCR, and endemic Victorian flavivirus (Murray Valley, Kunjin, Japanese encephalitis) PCR were negative. Autoimmune tests were also negative. Despite receiving empirical therapy for encephalitis—acyclovir, ceftriaxone, and benzylpenicillin—the patient continued to experience ongoing confusion, thrombocytopenia, and low-grade fevers. A repeat LP on day 9 of admission was sent for multiplex PCR using the BioFire FilmArray Meningitis/Encephalitis (ME) Panel. Results were unexpectedly positive for HHV-6 DNA. Ganciclovir (5 mg/kg Q12H) was commenced immediately. HHV-6 serology confirmed HHV-6 immunoglobulin (Ig) G without detectable IgM, consistent with reactivated infection, and at the conclusion of his ganciclovir therapy the patient was discharged from the hospital with resolution of fevers and thrombocytopenia at his cognitive baseline.

Given the complexity regarding the clinical significance of HHV-6 DNA in the CSF in immunocompetent individuals, the aim of this review was to evaluate the different methods by which a diagnosis of HHV-6 encephalitis is made and highlight the inconsistencies in diagnostic approaches to this condition.

## METHODS

We performed a literature review using Ovid to identify case reports of HHV-6 encephalitis in the immunocompetent adult population. The databases selected to search were MEDLINE and Embase classic + Embase. The keywords employed were “HHV-6” OR “human herpesvirus 6” OR “HHV-6,” AND “meningitis” OR “encephalitis” OR “meningoencephalitis” OR “meningo-encephalitis,” AND “immunocompetent” or “immuno-competent.” No time parameter was set in the search in order to capture as many case reports and case series as possible. Exclusions made during screening were records written in a non-English language without an English translation available, records with no full text available, and records in which it was evident in the title/abstract that the case pertained to a non-HHV-6 infection or non-meningoencephalitis presentation. Papers describing otherwise immunocompetent adults with type II diabetes mellitus were included in the literature review, as were patients who were given immunosuppressive medications such as corticosteroids during the course of their HHV-6 encephalitis infection, provided that symptoms of encephalitis preceded the administration of the immunosuppressive agent.

**Figure 1.**
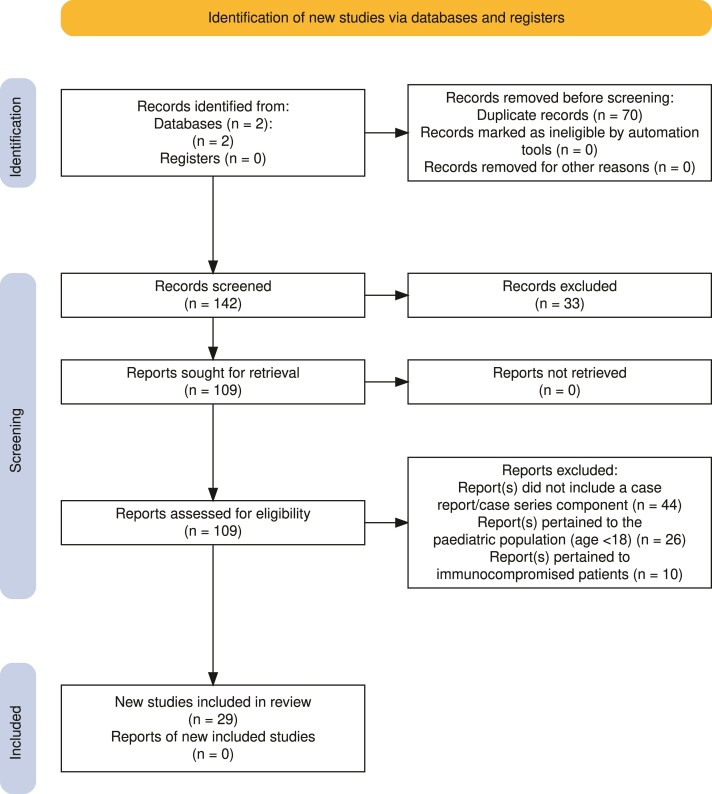
A PRISMA diagram outlining the literature search for HHV-6 encephalitis cases in the immunocompetent population [56]. Abbreviation: HHV-6, human herpesvirus 6.

A summary of the literature search is outlined in Figure 1.

## RESULTS

The 29 articles selected [3–31] all contain details of presentations of HHV-6 encephalitis in immunocompetent adults. Two articles [4, 11] included multiple HHV-6 encephalitis case descriptions, such that 34 cases of HHV-6 encephalitis in immunocompetent adults are discussed in the available literature. One case report identified in the literature search featured a positive HHV-6 DNA finding in the CSF, but ultimately did not conclude that HHV-6 was the definitive cause of the patient’s presentation [26]. This case is included in our discussion as it provides valuable insight into the complexity of making a diagnosis of HHV-6 encephalitis; however, it is not included in the analysis of cases diagnosed as HHV-6 encephalitis, given the acknowledgement in the article that HHV-6 encephalitis cannot be confirmed due to the additional finding of chromosomally integrated HHV-6, discussed later. We describe an additional case in which a diagnosis of HHV-6 encephalitis was made in a metropolitan hospital in Australia, bringing the total cases reviewed to 35.

## DISCUSSION

### Presentation of Illness

Regardless of the causative pathogen, viral encephalitis is known to present in a rather nonspecific fashion, and hence a broad range of symptoms ought to raise concern for viral encephalitis. Common presentations for all viral encephalitis include fever, headache, altered conscious state, confusion, seizures, and varied focal neurological signs on examination [32]. Table 1 outlines the signs and symptoms in the reported diagnoses of HHV-6 encephalitis, highlighting the variability of presentations that have led clinicians to explore the possibility of HHV-6 encephalitis.

**Table 1.** Presenting Signs and Symptoms Described in HHV-6 Encephalitis in Immunocompetent Adults in the Literature and Number of Cases With Each Described Sign/Symptom

Presentation	No. of Cases With Symptom
Confusion/disorientation	19
Subjective or objective fever	15
Seizure	13
Headache	12
Abnormal neurological exam including loss of power, hyperreflexia, fasciculations, ataxia, & paresthesias	10
General malaise	8
Nausea/vomiting	7
Other visual disturbance: loss of vision, blurred vision	6
Personality changes inc. irritability	5
Nuchal rigidity	5
Chills	4
Speech difficulty	3
Photophobia	3
Sore throat/URTI symptoms	2
Myalgia	2
Diarrhea	2
Anorexia	2
Back pain	2

Abbreviations: HHV-6, human herpesvirus 6; URTI, upper respiratory tract infection.

### Diagnosis

Much of the discourse surrounding HHV-6 encephalitis, particularly in immunocompetent individuals, is framed around the difficulty of establishing a certain diagnosis of encephalitis with HHV-6 as the causative pathogen. Almost all individuals are likely to have been exposed to HHV-6, symptomatically or not, before adulthood, and hence clinical presentations of HHV-6 in adulthood are likely the result of viral reactivation [1, 2]. HHV-6 latency occurs in monocytes and lymphocytes [33]. Asymptomatic viral reactivation is possible, so it is crucial that a positive HHV-6 DNA finding in the CSF be matched with symptoms that align with encephalitis, particularly in cases where monocytes and lymphocytes are also identified in the CSF [34]. All cases discussed in this article demonstrated clinical features of encephalitis that warranted further investigation.

Another point of diagnostic difficulty is that the HHV-6 genome is integrated into the host genome in ∼1% of the immunocompetent population and is passed on in a Mendelian inheritance pattern [35]. This is termed chromosomally integrated HHV-6 (ciHHV-6), and individuals with this condition have HHV-6 DNA present in the telomeres of all nucleated cells [36]. Hence, it is possible to receive a false-positive PCR result for active HHV-6 infection from a CSF sample containing any nucleated cells in an individual with ciHHV-6. A number of laboratory findings may point to an individual having ciHHV-6, including the presence of HHV-6 DNA indicated by PCR in hair follicles and high concentrations of HHV-6 DNA by quantitative PCR in the whole blood and serum [37]. A high concentration of HHV-6 DNA in serum has been defined as >3.5 log_10_ copies/mL (>3162 copies/mL) based on mean data of patients with established ciHHV-6, while >6.0 log_10_ copies/mL (>1 000 000 copies/mL) is considered high in whole blood samples [38]. Defining clear HHV-6 DNA copy counts in the CSF that may denote ciHHV-6 is a more challenging task, especially if the CSF contains leukocytes, as is often the case in infection. The presence of 1 viral copy per CSF leukocyte is highly suggestive of ciHHV-6, as well as >4.0 log_10_ copies/mL (>10 000 copies/mL) in the CSF, based on data from 21 patients [38]. Fluorescence in situ hybridization (FISH) and analysis of the genomes of parents are both useful in determining the presence of ciHHV-6; however, both prove impractical in the clinical setting due to laboratory limitations and obtaining access to samples from parents [39, 40]. While considering the limitations, we report the diagnostic approaches taken in HHV-6 encephalitis cases in immunocompetent individuals (Table 2).

**Table 2.** Diagnostic Methods for HHV-6 Encephalitis in Immunocompetent Adults

Diagnostic Method	No. of Cases Utilizing Method	Additional Details
CSF PCR for HHV-6 DNA	14 [4, 6, 7, 9, 14, 16, 17, 21, 23, 28, 29]	…
Consecutive CSF PCR for HHV-6 DNA showing elimination of viral DNA after antivirals	1 [3]	…
CSF RT-PCR for HHV-6 DNA	1 [27]	…
Quantitative CSF PCR for HHV-6 DNA	6	110 000 copies/mL [12] 7200 copies/mL [30] 16 189 copies/mL [19] >999 999 copies/mL [22] 2959.5 copies/mL [18] 395 copies/mL [31]
Consecutive CSF PCR for HHV-6 DNA showing reduction in HHV-6 DNA copy count after antivirals	1	30 000 copies/mL day 3 of antiviral treatment, 4400 copies/mL day 31 of antiviral treatment [15]
Multiplex CSF PCR, including Argene Biosoft, BioFore, & New York Encephalitis Panel: total	9	…
Multiplex: Quantitative Herpes Consensus CSF PCR (Argene Biosoft)	4	18 600 copies/mL [11] 9993 copies/mL [11] 38 000 copies/mL [11] Copy number not specified [8]
Syndromic encephalitis/meningitis CSF PCR panel (BioFire)	3 [24, 25]	Includes the case described in case report component of this report
New York State Encephalitis Panel followed by quantitative CSF PCR for HHV-6 DNA	1	6200 copies/mL [20]
Brain biopsy and brain tissue PCR for HHV-6 DNA	2	HHV-6 early protein antigen in oligodendrocytes [3] HHV-6 antigen in glial cells and neurons + perivascular lymphocytic infiltrates & CSF PCR positive for HHV-6 DNA [10]
No PCR performed; diagnosis made on MRI findings & elevated serum HHV-6 IgG	1 [13]	…

Abbreviations: CSF, cerebrospinal fluid; HHV-6, human herpesvirus 6; IgG, immunoglobulin G; MRI, magnetic resonance imaging; PCR, polymerase chain reaction; RT-PCR, reverse transcription PCR.

Several case reports included details of blood and serum PCRs for HHV-6 DNA. These have been separated in 2 groups based on whether they fall over or under the suggested copy number threshold for ciHHV-6 [38].

- Quantitative HHV-6 DNA PCR performed in blood/serum, copy number below the suggested threshold for ciHHV-6: 2 [6, 18].
- Quantitative HHV-6 DNA PCR performed in blood/serum, copy number above the suggested threshold for ciHHV-6: 5 [12, 15, 19, 27, 30].

The clinical standard for diagnosis of viral encephalitis for most herpesviruses is a positive viral PCR, in conjunction with a clinical picture of encephalitis [41]. However, in the case of HHV-6 encephalitis, there is a clear need for clarification of the significance of a positive PCR with a quantitative HHV-6 PCR in blood/serum and in CSF, given the possibility of a ciHHV-6 false-positive result. Without access to the CSF analysis (namely the nucleated cell count) from the CSF sample that was specifically used in the quantitative PCRs, it is difficult to comment on the relevance of high levels of HHV-6 DNA in the CSF and whether this is more suggestive of ciHHV-6 than active infection, although it is noted that 7 cases fell above the suspicion threshold of >10 000 copies/mL in the CSF [11, 12, 15, 19, 22, 26]. As laboratory techniques such as next-generation sequencing (NGS) become increasingly available, identifying sites of HHV-6 chromosomal integration in patient samples to confirm ciHHV-6 will improve the robustness of a diagnosis of HHV-6 encephalitis made on the basis of a high quantitative PCR [42].

One of the cases reported positive HHV-6 DNA in a PCR of nail and hair follicles, demonstrating ciHHV-6, in the presence of symptoms consistent with encephalitis that improved with ganciclovir [26]. This case posed a diagnostic challenge and suggested that intrathecal antibody production against HHV-6 may be a telling point of differentiation between an incidental ciHHV-6 finding and active infection superimposed on ciHHV-6, with the negative HHV-6 IgG and IgM in the CSF making a diagnosis of HHV-6 encephalitis impossible to confirm [26]. Other studies have validated this suggestion, with 1 analysis demonstrating that elevated CSF HHV-6 IgG and IgM are more frequently present in cases of clinical encephalitis than in neurological diseases with other causes [43]. This suggests that determining intrathecal antibody production against HHV-6 in conjunction with CSF HHV-6 PCR may be a robust diagnostic method for HHV-6 encephalitis.

Cases in which serial PCRs took place and demonstrated a reduction in HHV-6 DNA copies following antiviral therapy provide evidence for HHV-6 encephalitis [3, 15]. Although the reduction in HHV-6 DNA copies could equally be representative of the resolution of an asymptomatic viral reactivation, correlating clinical improvement with a reduced viral load is highly suggestive of a true HHV-6 encephalitis infection.

There is utility in multiplex PCR tools such as the BioFire FilmArray Meningitis/Encephalitis (ME) Panel, particularly in cases where initial investigations have not yielded an etiology. While the multiplex PCR generally demonstrates a high clinical concordance of 98.4% for diagnosis of viral encephalitis, clinical discordance is highest in the diagnosis of HHV-6 encephalitis; 76.9% of HHV-6-positive CSF samples from immunocompromised and immunocompetent patients were determined to not demonstrate true HHV-6 encephalitis [44]. This discordance is likely a result of latency, asymptomatic viral reactivation, and ciHHV-6 [34, 45]. As multiplex PCR assays become more available, it is important to recognize their value in clinical presentations that may be explained by uncommon pathogens, but also to consider the use of subsequent testing to determine the clinical significance of this finding.

As discussed above, methods of confirming a true HHV-6 encephalitis diagnosis that show promise include the use of serial quantitative CSF PCRs demonstrating a decrease in HHV-6 viral load in conjunction with clinical improvement while on HHV-6-specific antivirals, the assessment of intrathecal antibody production against HHV-6, and the evaluation of the possibility of ciHHV-6 using methods including FISH and parental genome analysis. Ultimately, however, the limited examples of the use of these methods in the literature restrict the conclusions that can be drawn regarding the certainty of making a diagnosis based on these investigations. This highlights the need for further research into accurate and feasible diagnostic methods for HHV-6 encephalitis.

### Management

Establishing a treatment protocol for HHV-6 encephalitis for immunocompromised and immunocompetent populations alike has proved to be a challenge for clinicians. Most existing data surrounding effective treatment come from the post-transplant HHV-6 encephalitis population as they face much of the burden of disease. Intravenous (IV) ganciclovir and foscarnet are both proposed as first-line antiviral agents in this population due to their strong in vivo action against the virus and positive clinical results from the use of these agents [46–49]. The recommended antiviral doses for HHV-6 encephalitis following hematological stem cell transplant are 5 mg/kg Q12H for ganciclovir and 90 mg/kg Q12H for foscarnet [48]. Table 3 outlines the antiviral agents used in each case of HHV-6 encephalitis, the duration of therapy, and the clinical outcomes.

**Table 3.** Treatment Choice and Therapy Duration in Cases of HHV-6 Encephalitis in the Immunocompetent Population

Case	Therapy	Duration of Therapy	Outcome
[3]	Empirical antibiotics + IV acyclovir	Not described	Recovered with nil sequalae
[4] ×4 patients	Not described	Not described	Not described
[5]	IV acyclovir as HHV-6 not identified until postmortem	5 wk (until death)	Rapidly progressive blindness, vomiting, seizures, and then decreased level of consciousness; died of pulmonary embolism in week 5 of hospitalization
[6]	IV acyclovir 500 mg Q8H as targeted HHV-6 therapy	10 d	Recovered with nil sequalae
[7]	IV ampicillin, cefotaxime, acyclovir empirically; HHV-6 identified day 9, management not changed	14 d (until death)	Progressive decline in cognition, coma; died of pulmonary embolism day 14 of hospitalization
[8]	IV acyclovir (750 mg Q8H) empirically, HHV-6 not identified until postmortem	10 d IV therapy, another 5 mo in hospital	Neurological symptoms persisted; patient died 5 mo later of respiratory arrest
[9]	Cidofovir & probenecid until adverse reaction occurred, then ganciclovir (5 mg/kg Q12H)	6 d cidofovir & probenecid, 15 d ganciclovir	Represented 1 mo later due to EBV tonsillitis, otherwise well
[11]38F	IV acyclovir, then oral valacyclovir managing presumed HSV encephalitis; represented 6 wk later and required further IV acyclovir followed by oral valganciclovir once HHV-6 identified	21 d IV acyclovir therapy followed by oral tail; once HHV-6 identified, 6 wk oral valganciclovir	Ongoing neurological deficits 1 y later
[11]66F	IV acyclovir for possible HSV encephalitis, HHV-6 identified after discharge	4 d	Ongoing concentration difficulties, fatigue, and a slight tremor
[11]20F	Not described	22 d as an inpatient	Not described
[12]	IV ganciclovir (375 mg Q12H)	13 d	Neuropsychological deficits (mainly amnesia, apraxia, and aphasia) persisted for weeks but eventually resolved
[10]	Empirical cephalosporin + fluconazole, no antivirals started	…	Died due to progression of lethargy into coma
[13]	IV acyclovir	Unclear antiviral duration, 51 d of hospitalization	Recovered with nil sequelae
[14]	Empirical therapy only (IV therapy with vancomycin; 1 g daily), ceftriaxone (2 g every 12 h), ampicillin (2 g every 6 h), and acyclovir (10 mg/kg every 12 h)	Unclear of exact duration	Died later that year following discharge, death related to cognitive and functional decline
[15]	IV foscarnet & IV ganciclovir dual therapy, then ganciclovir monotherapy, then oral valganciclovir tail	26 d dual therapy, 2 mo oral therapy	Hospital admission complicated by coma, but made near full recovery following targeted therapy
[16]	Oral valganciclovir 900 mg BD	6 wk	Persistent mild feet paresthesia
[17]	IV acyclovir with the following scheme (10 mg/kg 3 times daily for 7 d and 800 mg for the following 7 d)	…	Almost complete recovery
[19]	IV foscarnet, then ganciclovir due to adverse reaction to foscarnet	21 d total	Recovered with nil sequalae
[18]	IV ganciclovir (5 mg/kg every 12 h)	14 d	Recovered with nil sequalae
[20]	IV foscarnet	22 d	Patient palliated and died
[21]	IV acyclovir	10 d	Recovered with nil sequelae
[22]	IV ganciclovir 2.5 mg/kg Q12H, then oral valganciclovir 900 mg BD	16 d IV therapy followed by oral tail	Represented 3 wk postdischarge with neurological symptoms requiring 9 mo IV ganciclovir followed by oral tail; nil sequelae since then
[31]	Empirical: intravenous vancomycin, ceftriaxone, metronidazole, and ampicillin; no specific antivirals	…	Discharged to hospice
[23]	IV ganciclovir	14 d	Recovered with nil sequelae
[27]	IV ganciclovir 5 mg/kg Q12H, then oral valganciclovir 900 mg BD, then 900 mg daily	10 d IV therapy, 4 d BD oral therapy, 7 d daily oral therapy	Recovered with nil sequelae
[28]	IV ganciclovir and IV oseltamivir	14 d ganciclovir followed by 4 d oseltamivir	Recovered with nil sequelae
[29]	IV ganciclovir	15 d	Hospital admission complicated by MSSA pneumonia, nil complications related to encephalitis
[24]	IV ganciclovir (250 mg every 12 h, 5 mg/kg)	Unspecified	Died after a month due to medical complications; NOTE: autoimmune encephalitis not investigated
[25]	IV ganciclovir, then valganciclovir	Several days IV, then several weeks oral for 21 total d therapy	Recovered with nil sequelae
[30]	IV ganciclovir 5 mg/kg daily followed by oral valganciclovir 450 mg daily	IV therapy timeline unclear, oral therapy 2 wk	Recovered with nil sequelae
Our case	IV ganciclovir 300 mg Q12H	18 d	Subsequently experienced seizures with no detectable HHV-6 DNA in the CSF

Abbreviations: CSF, cerebrospinal fluid; EBV, Epstein-Barr virus; HHV-6, human herpesvirus 6; HSV, herpes simplex virus; IV, intravenous; MSSA, methicillin-susceptible *Staphylococcus aureus*; PCR, polymerase chain reaction.

Several patterns are evident in the relationship between the antiviral management of HHV-6 encephalitis and clinical outcome. First, full recovery was most frequent among patients who received ganciclovir. Of the 14 patients who received IV ganciclovir, with or without a valganciclovir tail, 10 made a full recovery, 1 patient died, and 3 experienced residual neurological symptoms. It is important to note that autoimmune encephalitis was not investigated in the case that resulted in death, and no quantitative HHV-6 DNA PCR or ciHHV-6 testing was performed, so a false-positive HHV-6 encephalitis result masking an alternate cause for the patient's presentation must be considered [24]. Additionally, the patient experienced status epilepticus during their admission, which has been established as a poor prognostic indicator in HHV-6 encephalitis and other causes of encephalitis [24, 50]. One patient who experienced residual neurological symptoms following IV ganciclovir therapy was on the lowest dose of ganciclovir at 2.5 mg/kg Q12H for 16 days, followed by a valganciclovir tail. Given the successful treatment of most other patients on ganciclovir at higher doses, it is possible that this dosage was subtherapeutic. Additionally, this patient had a remarkably high viral load in the CSF (HHV-6 > 999 999 viral copies/mL), which raises suspicion for ciHHV-6. A full recovery was eventually achieved in this patient after 9 months of IV ganciclovir and an oral tail of valganciclovir [22]. Three other patients were given an oral valganciclovir tail after ganciclovir therapy, and all made a full recovery [25–27]. However, given the generally high success rate of IV ganciclovir as an agent against HHV-6 encephalitis, the value of extended oral valganciclovir therapy is unclear.

Dual therapy with ganciclovir and cidofovir, foscarnet, or oseltamivir yielded positive outcomes, with all 4 patients in this group making a full or near full recovery [9, 15, 19, 28]. Dual foscarnet and ganciclovir therapy is an established treatment protocol in HHV-6 encephalitis following allogeneic hematopoietic stem cell transplant (HCT), with the combination therapy resulting in lower mortality and fewer neurological sequelae than monotherapy of either drug in recipients with HHV-6 encephalitis, so its success in the immunocompetent population is unsurprising [51]. Interestingly, foscarnet as a sole HHV-6 antiviral was only used in 1 case, and the patient died during their hospital admission [20]. In this case, the patient's infection treatment was complicated by a new diagnosis of systemic lupus erythematosus that required high-dose steroids, an immunosuppressant that has been associated with increased HHV-6 reactivation and infection severity [52].

Duration of targeted HHV-6 IV antivirals (ganciclovir, foscarnet, or cidofovir) ranged from 13 days to 26 days. Guidelines for HHV-6 encephalitis patients with hematological malignancies and patients post–hematopoietic stem cell transplantation suggest at least 3 weeks of IV antiviral therapy in the form of foscarnet or ganciclovir, and that blood/serum or CSF PCR should demonstrate clearance of the HHV-6 virus [48]. In the immunocompetent population, at least 14 days of therapy at the recommended doses was sufficient in most cases to produce a clinical cure, with the cases that did not resolve within that time frame having been discussed above.

In light of the discourse surrounding the validity of HHV-6 diagnoses made on positive PCR findings, it is important to discuss the trend of outcomes in patients who did not receive antivirals with activity against HHV-6. Ten patients received acyclovir monotherapy. Four of these 10 cases resulted in death as a result of rapid functional decline in the setting of neurological symptoms or complications of extended intensive care hospitalization such as pulmonary embolism [5, 7, 8, 14]. Two patients experienced ongoing neurological deficits at the conclusion of their acyclovir therapy [11, 17]. The remaining 4 patients receiving acyclovir made a full recovery [3, 6, 13, 21]. Acyclovir has limited action against HHV-6 replication in vitro, and the mean acyclovir concentration required to inhibit viral replication and virus-induced cytopathicity is much higher for HHV-6 infections than in acyclovir-responsive viruses such as herpes simplex virus (HSV) [49, 53]. We note that that 71% (10/14) of patients who received ganciclovir as part of their therapy made a full recovery, compared with only 40% (4/10) of patients receiving acyclovir alone. Likewise, there was a 100% (2/2) mortality rate in cases where no antivirals were used at all. Despite the controversies surrounding the validity of an HHV-6-positive CSF PCR as a diagnostic measure for HHV-6 encephalitis, favorable outcomes were achieved in patient populations that were given treatment for HHV-6, even when nonquantitative CSF PCR in conjunction with clinical presentation was the primary diagnostic tool. This finding must be stratified with the risk of administering targeted treatment for HHV-6, including bone marrow suppression in the case of ganciclovir and the nephrotoxicity of foscarnet [54, 55], and with the risk that treating for HHV-6 encephalitis based on a CSF PCR finding, before excluding other etiologies for an encephalitic presentation, may result in clinical harm.

## CONCLUSIONS

Evidently, there is significant variability in the diagnostic methods utilized by clinicians to make a diagnosis of HHV-6 encephalitis in immunocompetent adults. Diagnosis of HHV-6 encephalitis is complicated by the possibility of asymptomatic viral reactivation and ciHHV-6. As such, diagnosing this condition is nuanced, and caution should be taken when making a diagnosis of HHV-6 encephalitis in immunocompetent adults to reduce the possibility of a false-positive result, which may misguide treatment. Confirming viral load with a quantitative PCR and demonstrating a reduction in viral copies in conjunction with clinical improvement once appropriate antivirals are administered, measuring intrathecal antibody production against HHV-6, and establishing the presence or absence of chromosomally integrated HHV-6 DNA can provide strength to a diagnosis; however, there is no clear consensus on the most appropriate diagnostic method for this condition based on the current literature. Despite this diagnostic dilemma, our review of the literature demonstrates that patients who were treated for HHV-6 encephalitis had more favorable clinical outcomes than those who did not receive treatment. We conclude that treatment for HHV-6 encephalitis should be given without delay if the disease is suspected based on a clinical presentation consistent with encephalitis and qualitative PCR findings; however, quantitative HHV-6 testing, CSF serology, and further workup for alternative causes of encephalitis including autoimmune encephalitis should not cease based on the initial finding of HHV-6 DNA in the CSF.
