# Correction to: Diagnostic Dilemmas: A Review of Reported Cases of Human Herpesvirus 6 Encephalitis in Immunocompetent Adults

**DOI:** 10.1093/ofid/ofae715

**Published:** 2024-12-12

This is a correction to: Gemma Webb, Mei Yen Michelle Leong, Emma Bishop, Marjoree Sehu, Diagnostic Dilemmas: A Review of Reported Cases of Human Herpesvirus 6 Encephalitis in Immunocompetent Adults, *Open Forum Infectious Diseases*, Volume 11, Issue 9, September 2024, https://doi.org/10.1093/ofid/ofae501

In the originally published version of the manuscript, in the “screening” section of the PRISMA diagram, the numbers outlined were errored.

Figure 1 should read:

**Figure:**
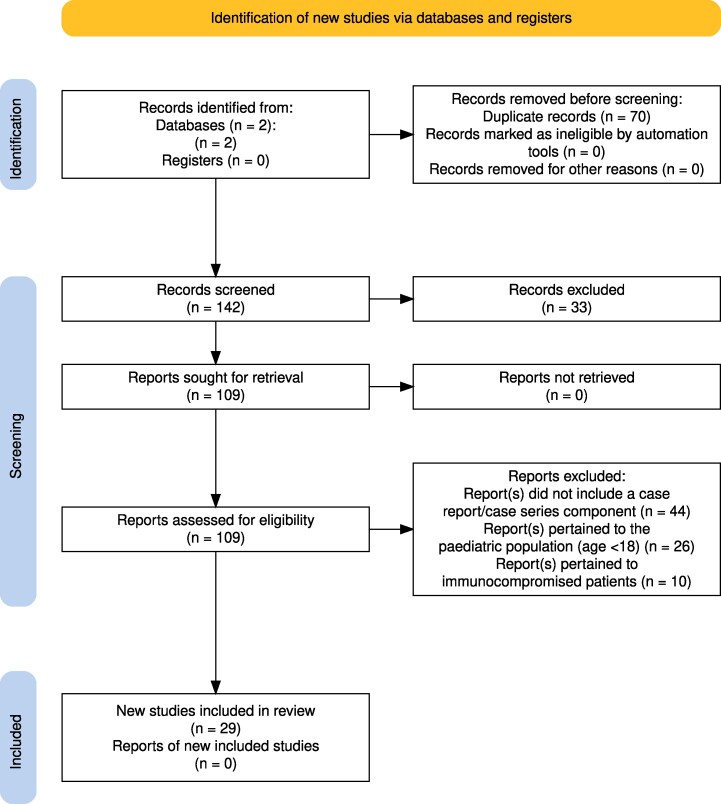


instead of:

**Figure:**
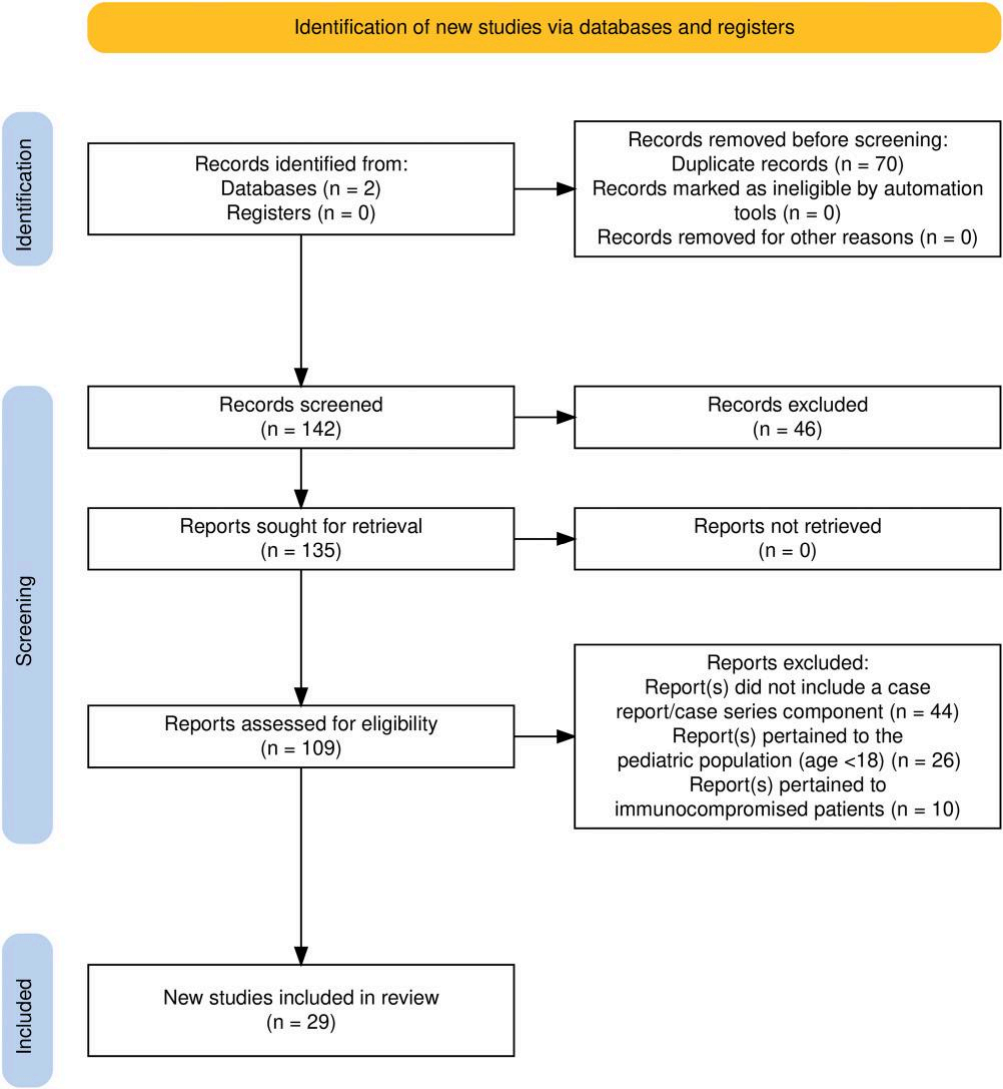


The emendation has been made to the article.

